# Mucormycosis of maxilla following tooth extraction in immunocompetent patients: Reports and review

**DOI:** 10.4317/jced.53655

**Published:** 2018-03-01

**Authors:** Kumar Nilesh, Aaditee V. Vande

**Affiliations:** 1 MDS. (Oral & Maxillofacial Surgery), Professor, Department of Oral & Maxillofacial Surgery, School of Dental Sciences, KIMSDU, Karad, India; 2 Post-graduate student, Department of Prosthodontics, School of Dental Sciences, KIMSDU, Karad, Maharashtra, India

## Abstract

Mucormycosis is a rare, fulminant, rapidly spreading fungal infection, which usually affects patient with underlying immune deficiency. If not managed promptly, the disease is characterized by progressive necrosis and is often fatal. A review of English literature shows that only fourteen cases of mucormycosis have been reported after tooth extraction. This paper highlights two cases of mucormycosis subsequent to tooth extraction in healthy adult patients. This first patient presented with an oroantral fistula and extensive maxillary necrosis. Whereas the second case was localized and presented as non-healing extraction socket with alveolar necrosis. This adds two more cases of this rare and serious complication of tooth extraction, to the present literature.

** Key words:**Fungal, infection, zygomycosis, exodontia, complication, jaw, necrosis.

## Introduction

Mucormycosis is a rare opportunistic infection invariably affecting immunocompromised patients. The organism implicated to cause mucormycosis is a saprophytic fungus, mainly rhizopus or mucor. It is the most deadly and rapidly progressive form of fungal infection affecting humans (1).

Clinical presentation of mucormycosis depends upon the site of entry of micro-organism and the organ systems involved. The most common form includes rhinocerebral, which involve the nose, paranasal sinuses, orbits and central nervous system. Other forms of mucormycosis are cutaneous, gastrointestinal, pulmonary and disseminated (2). Oral mucormycosis is usually caused by inhalation of spores or direct contamination of open oral wound. Oral mucormycosis affecting immunocompromised patients, mainly diabetes mellitus has been reported in literature (3). However those occurring subsequent to tooth extraction are rare (4). In view of the serious and potentially fatal complication of tooth extraction, this paper reports two such cases of mucormycosis presenting as oroantral fistula and non-healing extraction socket respectively, in healthy adult patients. The findings of the present cases are correlated with previously reported cases in English literature.

## Case Report

**Figure 1:**
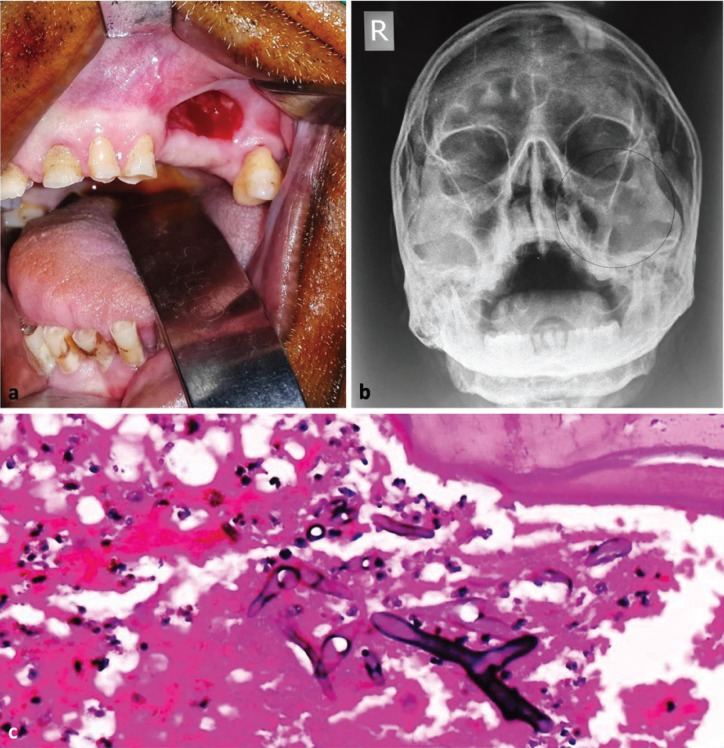
[Case 1] (Clinical presentation of the disease as oroantral fistula (a); Water’s view radiograph showing involvement of left maxillary sinus (b). Photomicrograph (H & E stained section; 40X magnification) showing broad and aseptate fungal hyphae with area of necrosed bone (c).

Case Report 1:

A 52 years old male patient, farm labourer by occupation presented with complaint of escape of fluid from nose after taking liquids and foul smell from mouth since past one week. Patient gave history of multiple teeth extraction one month earlier at a local private clinic. No contributory medical and family history was reported. Intraoral examination revealed an area of dehiscence over left maxillary alveolus with an oroantral fistula (Fig. 1a). Escape of water from left nostril after oral intake was demonstrated clinically. The left maxillary premolars and 1st molar were missing, confirming the history of previous extraction. Water’s view radiograph was advised to study the maxilla and the maxillary antrum. The radiograph showed destruction of left maxillary bone extending superiorly to the infraorbital rim and laterally to the zygomatic bone. Areas of radiopacity were evident within the left maxillary antrum, suggestive of sequestrum (Fig. 1b). Based on the clinical and radiological findings the diagnosis of maxillary osteomyelitis causing oroantral fistula was given. Gingival and bone incision biopsies were taken for histopathological study. The microscopic evaluation revealed broad and aseptate fungal hyphae within the area of necrosed bone (Fig. 1c). Based on the findings, a final diagnosis of maxillary osteonecrosis secondary to mucormycosis was established. Patient was recalled for hospitalization and intravenous antifungal therapy (amphotericin B; 0.8mg/kg/day for 4 weeks) was immediately started after serum urea and creatinine levels were found to be within normal range. Routine blood investigations, including serum glucose and complete haemogram showed no deranged values. Laboratory examination revealed no underlying immune deficiency.

**Figure 2:**
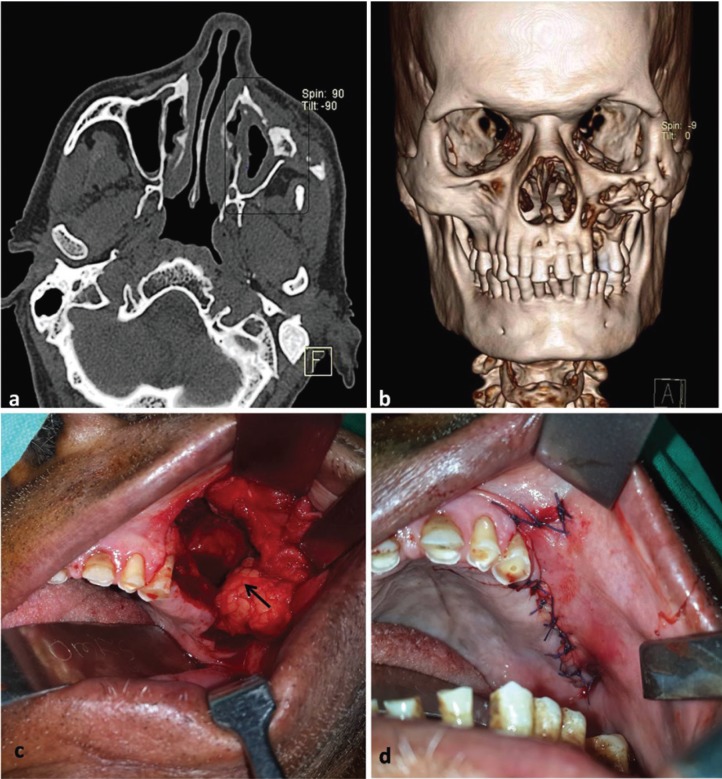
[Case 1] CT scan (axial section) showing thickening of antral lining and destruction of anterior wall of maxilla (a); Three dimensional formatted CT image showing involvement of left maxilla (b). Intraoperative pictures showing surgical debridement and removal of the sequestrum with the buccal fat pad mobilized into the defect (arrow) (c); closure of the oroantral communication (d).

The patient was subjected to computed tomography (CT) scan in order to study the extent and location of disease. Sectional views of CT scan showed thickening of left maxillary antrum lining, with destruction of anterior maxillary wall (Fig. 2a). Three dimensional formatted CT image showed destruction of anterior maxillary wall extending antero-posteriorly from the lateral nasal wall to the zygomatic bone and supero-inferiorly from the maxillary alveolus to just below the infraorbital rim. Area of loose bone was seen within the lesion suggestive of bony sequestrum (Fig. 2b).

Patient was prepared for surgical debridement and sequestrectomy along with closure of oroantral fistula, under general anesthesia. A written informed consent was taken for the same. Buccal mucoperiosteal flap was raised after excision of the fistula lining. The maxillary bone was exposed and the sequestrum removed. Debridement of maxillary antrum was done to remove the inflamed sinus lining, followed by copious irrigation with antiseptic solution. Pedicled buccal fat pad was mobilized by blunt dissection and used to close the defect posteriorly. The buccal mucoperiosteal flap was then advanced palatally over the buccal fat pad to attain two layered closure of oroantral communication (Fig. 2c,d). The patient showed uneventful recovery and was kept on regular recall visits. At 6 months follow-up patient did not show any further progression of the disease.

**Figure 3:**
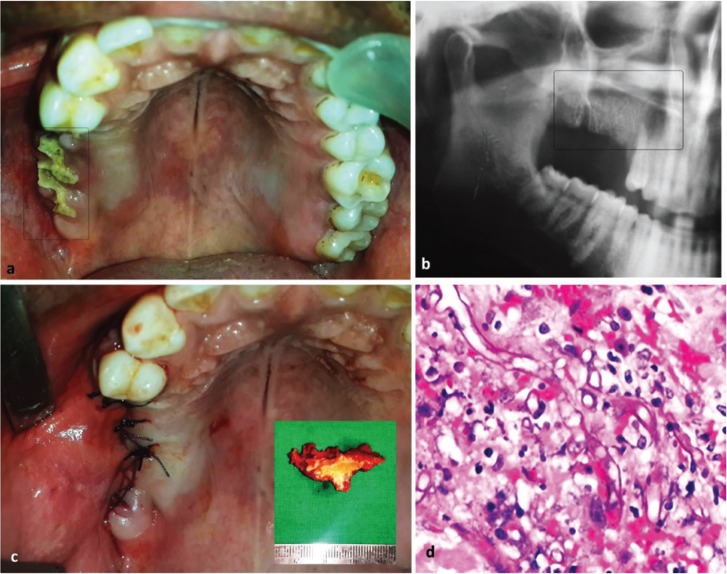
[Case 2] Clinical presentation as necrosed alveolar bone at site of extraction (a); Orthopantamogram showing of the involved region (b). Intra-operative photograph showing closure of the surgical site after removal of the necrosed alveolar bone (inset image) (c). Photomicrograph (H & E stained section; 100X magnification) showing aseptate, thin walled fungal hyphae with irregular contour (d).

Case Report 2:

A 37 years old male patient reported to our clinic with complaint of pain over upper right posterior region of jaw since past 2 weeks. Patient gave history of extraction of right upper right molars about six weeks back at a private dental clinic. The extraction was non-traumatic and the immediate post-extraction period was uneventful. No contributory medical and family history was reported. Intraoral examination showed dehiscence of mucosa over right maxillary alveolus. The crestal alveolar bone was exposed and appeared yellowish-white with no bleeding on probing (Fig. 3a). Orthopantomogram was advised, which showed missing mandibular right posterior teeth, with empty extraction sockets, indicative of recent extraction. The floor of the right maxillary sinus, in-relation to the apical aspect of the extraction sockets could not appreciated (Fig. 3b). Routine blood investigations were within normal limit. No underlying immune deficiency was evident on laboratory examination. Patient was prescribed oral antibiotics (Tablet Amocicillin 500mg + Potassium Clavulanate 125 two times a day) and nasal decongestant. Excision of the necrosed alveolar bone with closure of the defect with buccal advancement flap was planned and executed under local anesthesia (Fig. 3c). A written informed consent was taken for the same. The excised specimen was submitted for histopathological evaluation. Microscopic study of the necrotic alveolar bone showed presence of broad aseptate, thin walled fungal hyphae (Fig. 3d). Based on the presentation and histological findings, diagnosis of oral mucormycosis subsequent to tooth extraction was given. Patient was admitted and put on intravenous antifungal therapy (amphotericin B; 0.8mg/kg/day for 3 weeks). Periodic monitoring of serum urea, creatinine and renal function test were done, during antifungal therapy. There was no further progression of the disease and on four month follow-up visit patient showed satisfactory healing.

## Discussion

Mucormycosis is a rare opportunistic fungal infection caused by mucorales. It was first reported in humans by Paultaufin in 1885 (2). It is also known as zygomycosis or phycomycosis. Three common genera of mucorale which cause this disease in human include rhizopus, rhizomucor and absidia. Rhizopus accounts for 90% of cases involving head and neck region. These fungi exist in natural environment including soil, air, food, composite piles, and animal excreta and play role in decomposition. These fungal spores may be inhaled, ingested or may enter human body through open wound. Mucorales have been cultured from the oral cavity, nasal passage and pharynx of healthy individuals without any clinical signs of infection. Invariably this disease manifests, when the organisms affect an immunocompromised patients.

Angioinvasion of mucorales and its spores into the blood vessels lead to the formation of thrombus, which causes progressive necrosis of associated hard and soft tissues. The most common form of this disease in maxillofacial region is rhinocerebral mucormycosis, with widespread involvement of oral cavity, maxilla, palate, nose, paranasal sinuses, orbits and central nervous system. Early symptoms of this disease include facial cellulitis, periorbital edema and nasal inflammation, followed by widespread tissue necrosis. Failure of prompt medical and surgical intervention may lead to cerebral spread, cavernous sinus thrombosis, septicemia and multiple organ failure lending to high morbidity and mortality (3). The cases reported in this paper presented with a localized form of oral mucormycosis affecting immunocompetent patients, after tooth extraction.

Search of English literature from PubMed database, using combination of terms; mucormycosis, zygomycosis, extraction, exodontia, and maxillary necrosis revealed 34 titles, of which only eight cases were reported to be associated with tooth extraction. References of these papers were further scrutinized and additional five titles were identified. In total 13 papers (total of 14 cases) on mucormycosis secondary to tooth extraction were reviewed for demographic details, clinical presentation, extent of involvement, treatment provided and outcome ( Table 1,  Table 1 continue) (4-16). These cases were reported from all over the world, with 7 cases (50%) from India and 2 cases (14%) from USA. This unique distribution across the globe involving both developed and developing countries can be explained by relative lack of medical health care facility and more number of immunocompromised patients in developing countries like India. Whereas reports from developed country like USA can possibly be attributed to presence of multiethnic population (17). In this review, males were more commonly affected than females (in ratio of 13:5). While age of patient ranged from 14 to 74 years (mean of 52.21 years). The underlying condition predisposing this fungal infection included diabetes mellitus (8 cases), leukemia on chemotherapy (1 case), chronic obstructive pulmonary disease treated with steroid therapy (1 case) and diabetes mellitus along with leukemia (1 case). This finding was consistent with the fact that mucormycosis affect patients with compromised immunity. According to literature, 40-50% of patients suffering from mucormycosis have diabetes mellitus as a predisposing factor (3). Acidosis in diabetes mellitus compromises the phagocytic ability of white blood cells thereby affecting the host immunity. Interestingly among the cases reviewed in this paper, three were reported in patients with no immunocompromised condition (10,11,15). This finding was consistent with our cases. According to Mignogna M.D. *et al.* (17), mucormycosis affecting healthy individuals can be due to the role of local factors in pathogenesis of this disease. Local factors like surgical trauma from tooth extraction may compromise the local vascularity, as well as provide a portal of entry to the microorganisms. Tooth decay with periapical infection or periodontitis, which invariably are the most common cause of tooth removal, may further lower the local host defense mechanism. In the present review, extraction of maxillary posterior teeth was most commonly associated with this disease, accounting for 85% of all cases. While extraction of maxillary anterior teeth contributed for one case and mandibular molar for two cases (6,13). High propensity of association of mucormycosis with extraction of maxillary posterior teeth can possibly be due to their proximity to the maxillary sinus, which often get involved when fungal spores are inhaled through nasal route.

**Table 1:**
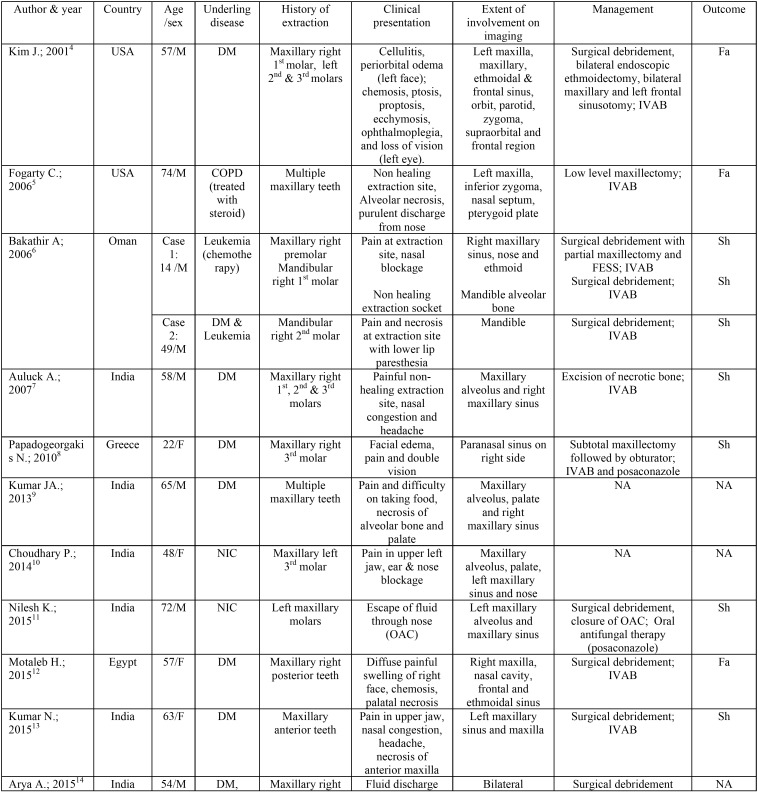
Review of previously reported cases of mucormycosis after tooth extraction.

**Table 1 continue:**
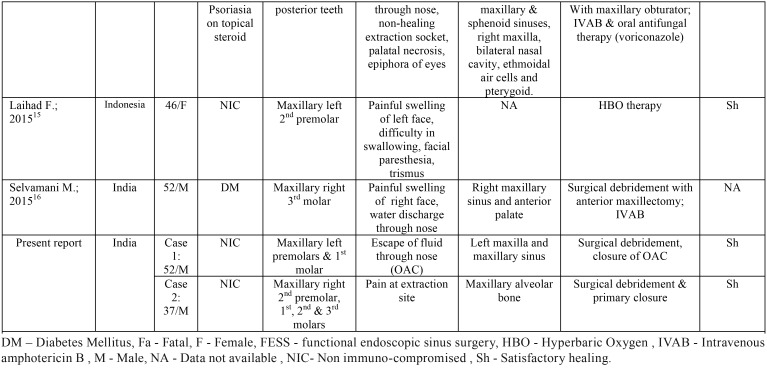
Review of previously reported cases of mucormycosis after tooth extraction.

Clinical features of the reviewed case included, non-healing extraction site (5,6,7,8,14,15,16), edema of face (4,12), alveolar bone necrosis with palatal ulcer (5,9,12,13), nasal discharge/blockage (5,6,7,10,13), paresthesia of lower lip (6), facial paresthesia (15), trismus (15) and headache (7). Further spread of infection result in orbital involvement, causing chemosis (edema of conjunctiva), epiphora (excessive watering of eye), diplopia (double/blur vision), ptosis (drooping of upper eyelid), proptosis (protrusion of eye ball), ophthalmoplegia (paralysis of eye muscles), and vision loss (4,8,12,14). As the disease is rapidly progressive, imaging modalities like computed tomography and magnetic resonance imaging are useful tools to study the extent of necrosis, paranasal sinuses involvement, orbital and cerebral spread. In the present review, majority of the cases showed extensive involvement of maxillofacial skeleton (71% of cases). However, the cases presented, along with three of the reviewed cases manifested as localized disease involving only the maxillary alveolar bone and maxillary sinus (7,11,13), whereas one case was localized only to mandibular alveolus (6).

Mucormycosis require prompt management to prevent further spread and avoid fatal complications. Treatment includes immediate hospitalization and systemic antifungal therapy. Amphotericin B is the drug of choice in mucormycosis. Supportive therapy includes; fluid balance, nutritional supplements and correction of underlying immune deficiency. Surgical intervention is often required to remove the necrosed tissue. In the present review, surgical management included combination of one or more procedures like tissue debridement, maxillectomy, sinus exploration and curettage. Functional endoscopic sinus surgery and hyperbaric oxygen therapy has also been reported for treatment of mucormycosis (6,15). The cases presented were managed by local debridement and sequestrectomy. Oroantral fistula in the first case was closed using double layered pedicled buccal fat pad and buccal advancement flap. The outcome of management of mucormycosis often depends on immune status of individual, extent of spread, cerebral involvement and systemic dissemination. The review of cases showed satisfactory result in 11 cases (79%), whereas three patients (21%) showed fatal outcome. Early diagnosis and intervention reduces the extent of spread and limits the risk of serious complications.

## Conclusions

Mucormycosis is a rare fungal infection which can cause widespread necrosis of orofacial tissues in susceptible host. Though incidence of mucormycosis secondary to tooth extraction is extremely low, however when it occurs, may cause significant morbidity and mortality. Hence dental professionals must be aware of the possibility of this serious and fatal complication, so as to avoid unfavorable outcome in clinical practice.
